# An analytical approach to aggregate patient inflows to a simulation model over the radiotherapy process

**DOI:** 10.1186/s12913-021-06162-4

**Published:** 2021-03-08

**Authors:** Jesper Lindberg, Paul Holmström, Stefan Hallberg, Thomas Björk-Eriksson, Caroline E. Olsson

**Affiliations:** 1 Department of Radiation Physics, Institute of Clinical Sciences, Sahlgrenska Academy, University of Gothenburg, 413 45 Gothenburg, Sweden; 2 Department of Medical Physics and Biomedical Engineering, Sahlgrenska University Hospital, 413 45 Gothenburg, Sweden; 3 Regional Cancer Centre West, Western Sweden Healthcare Region, 413 45 Gothenburg, Sweden; 4 Department of Oncology, Institute of Clinical Sciences, Sahlgrenska Academy, University of Gothenburg, 413 45 Gothenburg, Sweden

**Keywords:** Simulation, System dynamics, Radiotherapy, Data input set, Patient inflow

## Abstract

**Background:**

In meeting input data requirements for a system dynamics (SD) model simulating the radiotherapy (RT) process, the number of patient care pathways (RT workflows) needs to be kept low to simplify the model without affecting the overall performance. A large RT department can have more than 100 workflows, which results in a complex model structure if each is to be handled separately. Here we investigated effects on model performance by reducing the number of workflows for a model of the preparatory steps of the RT process.

**Methods:**

We created a SD model sub-structure capturing the preparatory RT process. Real data for patients treated in 2015-2016 at a modern RT department in Sweden were used. RT workflow similarity was quantified by averaged pairwise utilization rate differences (%) and the size of corresponding correlation coefficients (*r*). Grouping of RT workflows was determined using two accepted strategies (80/20 Pareto rule; merging all data into one group) and a customized algorithm with *r*≥0.75:0.05:0.95 as criteria for group inclusion by two strategies (*A1* and *A2*). Number of waiting patients for each grouping strategy were compared to the reference of all workflows handled separately.

**Results:**

There were 128 RT workflows for 3209 patients during the studied period. The 80/20 Pareto rule resulted in 14/8/21 groups for curative/palliative/disregarding treatment intent. Correspondingly, *A1* and *A2* resulted in 7-40/≤4-36/7-82 groups depending on *r* cutoff. Results for the Pareto rule and *A2* at *r*≥85 were comparable to the reference.

**Conclusions:**

The performance of a simulation model over the RT process will depend on the grouping strategy of patient input data. Either the Pareto rule or the grouping of patients by resource use can be expected to better reflect overall departmental effects to various changes than when merging all data into one group. Our proposed approach to identify groups based on similarity in resource use can potentially be used in any setting with variable incoming flows of objects which go through a multi-step process comparable to RT where the aim is to reduce the complexity of associated model structures without compromising with overall performance.

**Supplementary Information:**

The online version contains supplementary material available at 10.1186/s12913-021-06162-4.

## Background

Radiotherapy (RT) is used for approximately 50% of cancer patients to cure the disease or to ameliorate associated symptoms [1, 2]. With a growing cancer incidence, demands on RT are increasing. One immediate problem is how to maximize the utilization rates of available resources whilst maintaining high treatment quality and staff satisfaction [3]. RT is one of the most complex disciplines of healthcare and understanding departmental responses to various changes can be challenging. Simulation models, as suggested by system dynamics methodology or other methods within the field of operations research (OR), can help to increase this understanding [4, 5]. To create such a model referral of patients to RT, which determines the input data format, is the first step of the RT process and must be thoroughly understood.

The RT process involves numerous tasks from treatment preparation to treatment delivery. The treatment is typically fractionated, *i.e.* given once daily during a 5-7 week period, and delivered using linear accelerators “*linacs*” [6]. Preparations are undertaken to assure that the desired treatment is given to the intended anatomical region. Different imaging modalities are used to obtain a 3D-image representation of the patient’s anatomy on which contours of the tumor to be treated and the organs that must be avoided during treatment are overlaid. Based on this information, a team of physicians, physicists, and nurses (specialized in radiation oncology or radiation physics) then decide the treatment setup and calculate the dose distribution. Each RT task needs to be coordinated in time with additional treatments such as surgery or chemotherapy with the aim to assure that the scheduled activities meet the need of each patient. The referral pattern of patients to RT largely impact if this is feasible given diagnosis- and treatment-intent-specific dependencies in tasks as well as the balance between existing workload and available capacity at the department.

When addressing patient referral patterns to an RT department from a modelling perspective with the ultimate aim to understand the overall structure, it may be challenging to keep the model parsimonious. Patients are referred to RT in inherently variable volumes [7]. Non uniformity can be expected with respect to time and with respect to composition of diagnoses and of treatment intents. Temporal variations mainly depend on availability of staff, both for clinical assessments as well as for diagnostics; these effects are to some extent predictable. Effects due to vacation periods during summer or longer holidays are also predictable with the number of referrals tending to increase before affected dates whilst decreasing to reach a steadier state after the period in question has passed. The composition of referrals has a more random structure in the short perspective but also tends to stabilize over time given a particular RT departments profile. Complicated patient cases typically demand non-standard approaches and such cases are often directed to larger hospitals for centralized care. The mix of cancer diagnoses and treatment intents at a particular RT department is therefore a consequence of the available treatments. Taken together, some treatments are common at certain RT departments but may not exist at all at others. In addition, the number of patients within a specific group needs to be acknowledged.

In order not to inflate the size of a simulation model more than necessary and make it difficult to build, analyze and understand, applying the 80/20 Pareto rule to the input data is one strategy [8]. For the current problem, this would mean to use the distribution of incoming referrals for 80% of the patients and rescale this volume to match the total number of patients. Another strategy could be to combine the remaining 20% of patients into one group. A drawback with these approaches is that effects for patients who are treated according to rare diagnoses may not be properly acknowledged. This is problematic if different operational or capacity policies involving them are to be tested. To compensate for this, reducing model complexity without changing effects by the overall input data could be achieved by grouping patients which are comparable from some aspect, *e.g.* which utilize the same RT resources to a similar degree.

OR methods in RT have historically mainly focused on resource planning and resource use for purposes such as optimizing staff allocation, scheduling of patients or understanding the RT process from a strategic perspective [9]. Work on OR models for RT done by Vieira et al. used the referral pattern of patients to RT for one month and, based on this, they could assume a daily patient volume based on the Poisson distribution with a mean number of patient arrivals corresponding to observed rates of a particular weekday [7]. Discrete-event simulations by Kapamara et al. used data for one year (2005), uniform and exponential probability distributions were used to estimate input values for various variables including number of patients and their characteristics [10]. Using the same discrete-event simulation computer package (Simul8 Corporation, Boston, MA, U.S.A.), Proctor et al. also designed a model to identify factors affecting how a patient moves through the RT department from initial consultation/referral to the last treatment fraction [11]. They used data from a two-linac department, also in the U.K. (1997/2001), to estimate the performance of the department with an increased level of demand. Patient characteristics including requirements of RT resources were determined from a probability (profile) distribution. These are some of only a few examples, which address the referral pattern of patients to RT in some detail, and to the best of our understanding it is unknown how different approaches to group patients of an RT department compare to each other in an OR setting.

In this work, we took an analytic approach to systematically quantify the input data to a sub-structure of a system dynamics (SD) model which mimics the preparatory steps of a seven-linac RT department in Sweden. This is one of the larger departments in this country, located at one of our university hospitals, and it offers treatments for all kinds of cancer diagnoses and treatment intents. Our aim was to identify an aggregated input data set, which reproduced and simulated effects according to existing patient referral patterns without having to handle each diagnosis and treatment intent separately. Understanding the effects by aggregating the input data for this problem will increase the knowledge about how to create future full-scale system dynamics models of the whole RT process or other systems with similar characteristics.

## Materials and methods

### Data

Patient data were retrieved from the Oncological Information System ARIA® (Varian Medical Systems, Inc., Palo Alto, CA, U.S.A.) during January 1^st^ 2015 to April 30^th^ 2016. All patients referred to RT at Sahlgrenska University Hospital, Gothenburg, Sweden during this period (70 weeks) were sorted according to cancer diagnosis, treatment intent, and utilization of departmental resources for the preparatory steps of the RT process. Patients who had been registered in the system during the investigated period, but where no treatments had been delivered, were also included.

Cancer diagnoses were sorted according to ICD-10 codes [12] and treatment intent was acknowledged as “curative” or “palliative”. Each diagnosis- and treatment-intent-specific care pathway was defined as a separate workflow with the types of resources needed to prepare such patients for RT (hereafter referred to as an RT workflow). Resource utilization rates were quantified based on percentage use of associated operations or tasks given the total number of patients referred to the RT workflow in question (later referred to as PPRCT, Percentage patients requiring capacity). Some patients may require more than one appointment, hence PPRCT can exceed 100%. Assessed tasks were categorized into: 1. *Positioning aid (Mould)*, 2. *Positron-emission tomography (PET), 3. Computed tomography (CT), 4.* M*agnetic resonance imaging (MRI)*, 5. *Target definition (TD)*, 6. *Treatment planning (TP),* and 7. *Patient quality assurance (QA)*.

### Statistics

Similarity between different RT workflows was calculated using pairwise comparisons between resource utilization rates as quantified by Mann-Whitney U tests and Pearson’s linear correlations (*r*). Absolute percentage differences were also calculated for each task and as a total summary metric over all tasks. RT workflows with quantitatively similar patterns, *i.e.* similar use of resources, were then identified and aggregated based on increasingly stronger correlation cutoffs (*r*≥0.75:0.05:0.95); a customized algorithm was developed to systematically investigate these as termination criteria for group inclusion (details on the algorithm available in Additional file 1). Two grouping strategies were investigated: 1. All elements correlate with one main element (*GroupingStrategy 1,* referred to as *A1* below) and 2. All elements correlated with each other (*GroupingStrategy 2,* referred to as *A2* below). To minimize potential effects by selected starting point for this procedure, random permutations of RT workflows were done 10 000 times/*r*-value cutoff. RT workflows were primarily required to have the same treatment intent to be paired, but effects by disregarding treatment intent were also investigated.

The data handling and the calculations were performed in Microsoft Excel (2016) or in MATLAB (MATLAB R2018a version 9.4.0.813654, The MathWorks Inc., Natick, MA, U.S.A.). *P*-values≤0.05 were considered to indicate statistical significance and *r*-values≥0.70 were considered to indicate strong correlations. Descriptive statistics were reported using mean and standard deviation or median and range, whichever most suitable based on the underlying data distribution. Performance between grouping strategies was quantified by absolute and relative differences at each time point with respect to a reference grouping strategy, which handled all workflows separately.

### Model and modelling scenarios

**Fig. 1:**
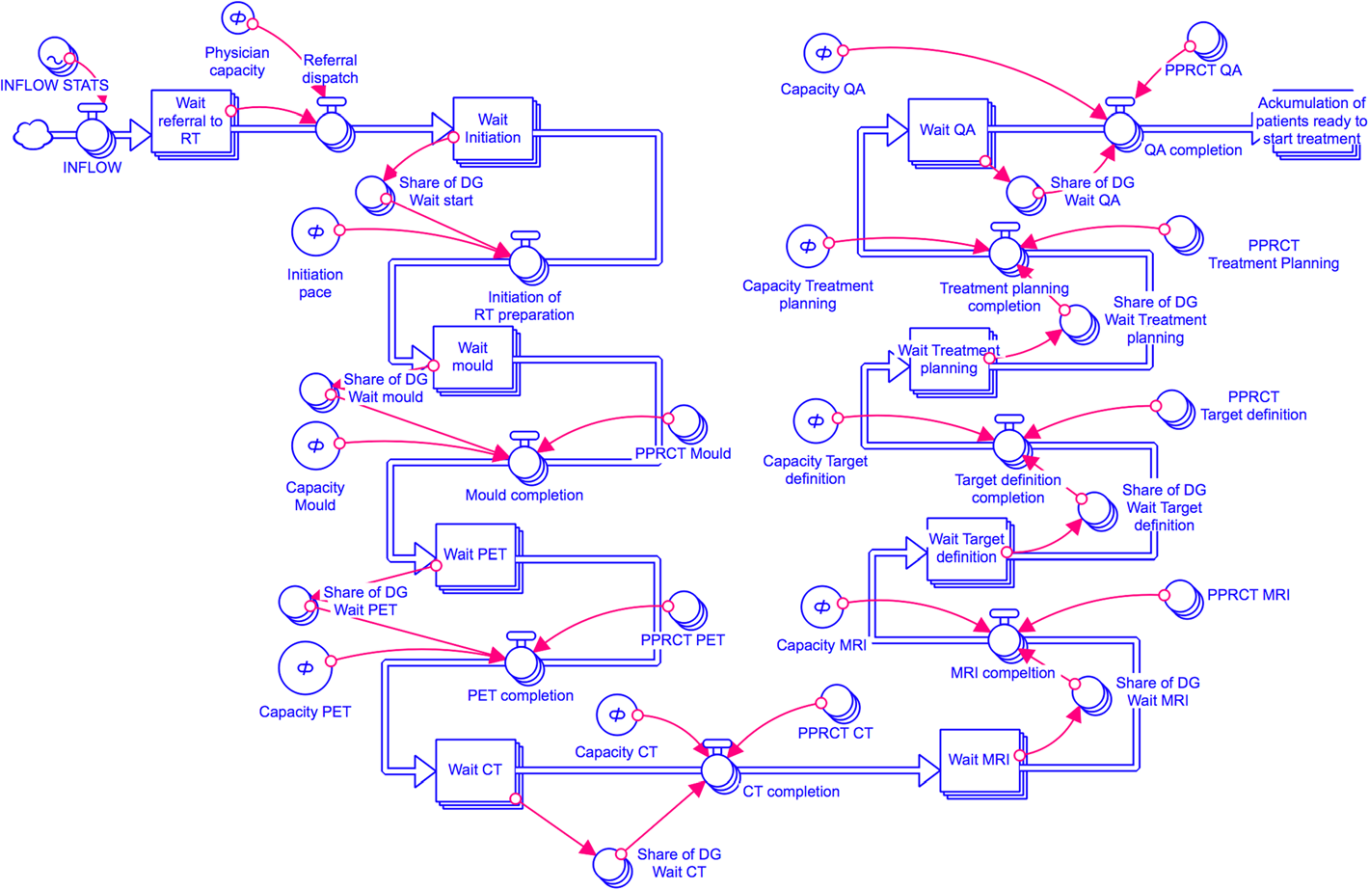
A sub-structure of a system dynamics model capturing the preparatory steps of the RT process built in over seven RT preparatory tasks evaluating number of waiting patients from scenarios defined by various capacity settings, combined with actual patient volumes and corresponding capacity demands. Note that the flow starts at the top left of the graph and finishes at the top right. If the workflow in question does not include a certain operation, the patients flow through this task without any capacity demand; this never happens at the boundaries of the model (referral and ready to start treatment). *Symbols*: Rectangles = points in the flow where patients accumulate; blue thick lines with valve symbol = route for patients transferring through a capacity-restricted operation; red fine lines with arrow head = transfer of information held in the circular symbol to be utilized in a computation at the point of the arrowhead. At each capacity restricted operation, two variables are combined to determine what flows through, capacity for the operation and the variable PPRCT x, which determines the percentage of the waiting patients who will be requiring capacity in operation x. *Abbreviations*: CT=Computed Tomography, MRI=Magnetic Resonance Imaging, PET=Positron Emission Tomography, PPRCT=Percentage patients requiring capacity, QA=Quality Assurance, and RT=Radiotherapy

We investigated the effects of using the different grouping strategies to aggregate RT workflows as input data to a sub-structure of a SD model capturing the preparatory steps of the RT process, which will be developed for system dynamics analysis of the whole RT process in future work. The present model provided the overview of patient flows and number of patients waiting through subsequent operational steps required to prepare the patients for RT. The input data were handled in weekly batches. The starting point of the proposed seven-step model was when a treatment decision was taken as a documented referral date to the RT department; the end point was when a date for completed QA was documented, confirming that the patient was ready to start treatment (Fig. 1; Additional file 1). Available resources at each operational step were calibrated to a same capacity setting which allowed for patient throughput at a rate not to build queues for the worst-case scenario by each treatment intent. The simulations were performed in Stella Architect (v.1.7.1 isee systems, Lebanon, NH, U.S.A.) and run on a MacBook Pro 2016 (MacOS v.10.13.1).

Model outputs were compared over all time points based on resulting number of patient waiting as a measure of the ability of each grouping strategy to match the number of patients waiting of the best-case scenario where all workflows were handled separately (*reference*). Aggregation of RT workflows by the customized algorithm are acknowledged by the grouping strategy number, *i.e.* A1 or A2, and utilization rate correlation coefficient cutoff, *i.e. 75, 80, 85, 90 or 95. A1_75*, therefore, refers to results by *A1* with cutoff at *r*=0.75. For comparison, all RT workflows were also grouped according to the Pareto rule (80% of workflows handled separately and the remaining 20% merged into one joint group; *Pareto_80/20)*. All RT workflows were also merged into one group as an estimate of the expected least representative worst-case scenario (*all-in-one*).

## Results

### Data overview

**Fig. 2:**
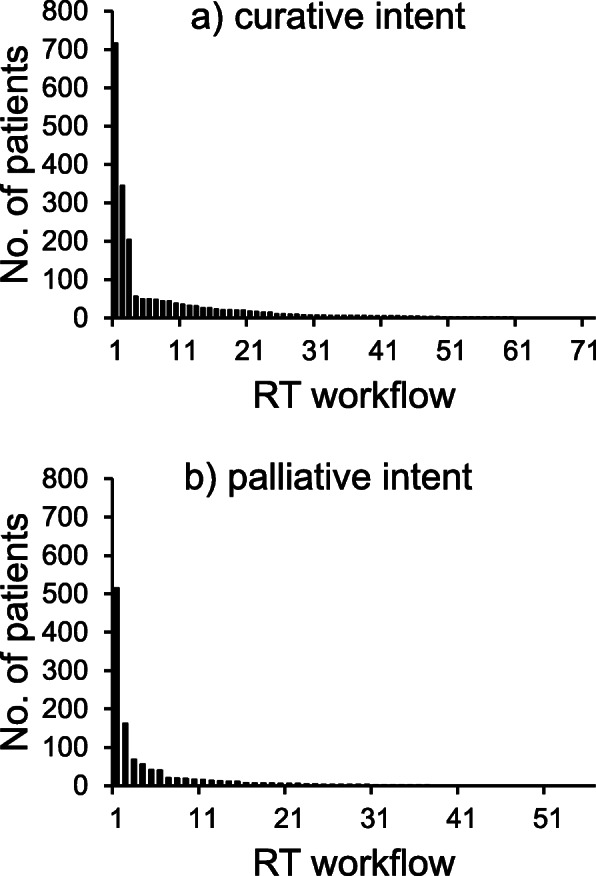
Number of patients included in each RT workflow for curative (**a**) and pallative (**b**) intent

In total, 3209 patients were referred to the RT department during the studied period. Of these, 2094 (65%)/1115 (35%) were planned for treatment with curative/palliative intent. There were 72/56 different cancer diagnoses resulting in 128 separate RT workflows, distribution of patients per RT workflow is showed in Fig. 2a-b. The majority of curative patients were to undergo a single treatment course (98%), but almost one of three palliative patients underwent more than one treatment course (32%). The treatments for five patients planned for seven treatment courses were cancelled although preparations for RT were completed.

### Similarity between workflows with and without consideration of treatment intent

For curative treatments, similarity between utilization rates of resources between RT workflows could generally not be ruled out (averaged minimum *p*=0.057). In two situations, however, candidates for grouping could be excluded (≥1 diagnosis/RT workflow; *p*<0.042). For the remaining comparisons, statistically significant correlations were generally strong (averaged median *r*=0.87, range: 0.78-0.97) and typically offered multiple candidates for grouping (median: 18 pairs; Table 1). The RT workflow for C61 (prostate cancer) showed no statistically significant correlations with any other workflow. The RT workflows for C24 (bile duct cancer) and L91 (hypertrophic skin disorder) correlated with only one other workflow. The average absolute percentage difference for total resource utilization rate for all curative RT workflows, calculated as median of all mean values, was 38% (range: 22-238%).

**Table 1:** Averaged statistically significant correlations for 72 RT workflows where patients were treated with curative intent at the Sahlgrenska University Hospital in Sweden during 2015-2016

r	Mean	SD^a^	Median	Min	Max
**Mean**	0.87	0.03	0.87	0.80	0.95
**SD**^a^	0.06	0.01	0.06	0.04	0.09
**Median**	0.87	0.05	0.87	0.78	0.97
**Min**	0.76	0.10	0.77	0.00	0.87
**Max**	0.95	0.12	0.98	0.00	1.00
**#**	18	9	15	0	35

Note that aggregations of the presented results are done for the r-values of each RT workflow over all RT workflows, so that the calculations in the first row relate to the mean values of all RT workflows, the calculations in the second row relate to all SD values, etc.

*Abbreviations*: *r* correlation coefficient, *SD* standard deviation, *#* number of potential candidates for grouping, *RT* radiotherapy

^a^SD excluded in calculations for groups with one element only

For palliative treatments, similarity between utilization rates of resources between RT workflows could, as for curative treatments, generally not be ruled out (averaged minimum *p*=0.1820). There was only one situation where candidates for grouping could be excluded (3 diagnoses; *p*<0.034). Statistically significant correlations between RT workflows were strong (averaged median *r*=0.90, range: 0.77-0.96) and offered several candidates for grouping (median 42 pairs; Table 2). The RT workflows for C56 (ovarian cancer) and C64 (kidney cancer) showed no statistically significant correlations with any other workflow. The average absolute percentage difference for total resource utilization rate for all palliative RT workflows was 20% (range: 8-95%).

**Table 2:** Averaged statistically significant correlations for 56 workflows where patients were treated with palliative intent at the Sahlgrenska University Hospital in Sweden during 2015-2016

r	Mean	SD^a^	Median	Min	Max
**Mean**	0.90	0.03	0.91	0.80	0.93
**SD**^a^	0.06	0.01	0.06	0.03	0.08
**Median**	0.90	0.04	0.91	0.77	0.96
**Min**	0.74	0.15	0.77	0.00	0.83
**Max**	0.95	0.19	1.00	0.00	1.00
**#**	42	13	46	0	51

Note that aggregations of the presented results are done for the r-values of each RT workflow over all RT workflows, so that the calculations in the first row relate to the mean values of all RT workflows, the calculations in the second row relate to all SD values, etc. *Abbreviations:* as in Table 1

^a^SD excluded in calculations for groups with one element only

The pattern of similarity between utilization rates of resources remained, irrespective of treatment intent, (averaged minimum *p*=0.073). There were five situations where candidates for grouping could be excluded. Correlations between RT workflows were strong (averaged median *r*=0.88, range: 0.78-0.97) and offered more candidates for grouping than either of the two treatment intents separately (median: 50 pairs; Table 3).

**Table 3.** Averaged statistically significant correlations for 128 workflows disregarding treatment intent for patients at the Sahlgrenska University Hospital in Sweden during 2015-2016

r	Mean	SD^a^	Median	Min	Max
**Mean**	0.87	0.03	0.88	0.80	0.94
**SD**^a^	0.06	0.01	0.06	0.00	0.09
**Median**	0.88	0.05	0.88	0.78	0.97
**Min**	0.77	0.02	0.76	0.75	0.88
**Max**	0.98	0.03	0.99	0.88	1.00
**#**	50	2.8	65	2	84

Note that aggregations of the presented results are done for the *r*-values of each RT workflow over all RT workflows, so that the calculations in the first row relate to the mean values of all RT workflows, the calculations in the second row relate to all SD values, etc. *Abbreviations:* as in Table 1

^a^SD excluded in calculations for groups with one element only

However, the RT workflow for C61 (prostate cancer) with curative treatment intent had no potential grouping candidate; remaining workflows had ≥2 candidates for grouping. Averaged absolute percentage difference for total resource utilization rate for all workflows was 17% (range: 8-100%)).

### Aggregating RT workflows

Using *A1*, where all elements in a RT workflow group were to correlate with one main element, and *r*≥0.75/0.80/0.85/0.90/0.95 resulted in a minimum of 7/9/12/18/28 groups for curative intent (Table 4, left). Corresponding figures for palliative intent were 4/4/5/7/12 groups and 7/10/13/21/34 groups when disregarding treatment intent. Within each group of similar characteristics, absolute percentage differences for resource utilization rates between included elements were on average at most 107/113/132/202/279% for curative intent, 39/37/39/38/63% for palliative intent, and 71/67/69/65/123% when treatment intent was disregarded.

**Table 4:** Number of groups and averaged within-group differences by the customized algorithm and correlation coefficient cutoffs for patients treated for either curative or palliative intent at the Sahlgrenska University Hospital in Sweden during 2015-2016

Strategy	***A1***										***A2***									
**Treatment intent**	**CURATIVE**																			
***r***	**0.75**		**0.8**		**0.85**		**0.9**		**0.95**		**0.75**		**0.8**		**0.85**		**0.9**		**0.95**	
	**No**	**∆%**	**No**	**∆%**	**No**	**∆%**	**No**	**∆%**	**No**	**∆%**	**No**	**∆%**	**No**	**∆%**	**No**	**∆%**	**No**	**∆%**	**No**	**∆%**
**Mean**	10.0	53.9	11.7	58.1	15.0	64.7	22.5	69.7	32.7	79.3	10.0	53.8	19.6	76.7	28.6	99.8	36.8	104.0	45.0	105.4
**SD**	0.9	14.0	0.8	14.8	1.1	16.0	1.2	25.9	1.3	48.8	0.9	14.3	1.1	21.4	1.2	28.9	1.2	44.5	1.3	62.6
**Median**	10.0	50.5	12.0	55.7	15.0	63.4	23.0	67.6	33.0	66.3	10.0	50.3	20.0	76.8	29.0	103.5	37.0	112.5	45.0	113.4
**Min**	7.0	25.7	9.0	22.9	12.0	23.1	18.0	16.8	28.0	0.0	7.0	26.7	15.0	21.5	24.0	9.6	33.0	2.7	40.0	0.0
**Max**	12.0	107.1	13.0	113.2	18.0	132.3	26.0	202.0	36.0	278.6	12.0	109.1	24.0	160.4	33.0	189.6	41.0	257.5	49.0	278.6
**Treatment intent**	**PALLIATIVE**																			
***r***	**0.75**		**0.8**		**0.85**		**0.9**		**0.95**		**0.75**		**0.8**		**0.85**		**0.9**		**0.95**	
	**No**	**∆%**	**No**	**∆%**	**No**	**∆%**	**No**	**∆%**	**No**	**∆%**	**No**	**∆%**	**No**	**∆%**	**No**	**∆%**	**No**	**∆%**	**No**	**∆%**
**Mean**	5.0	27.1	5.2	26.7	6.9	25.7	9.6	24.4	14.3	23.7	5.0	27.1	10.3	22.4	18.1	17.5	27.6	18.4	39.0	14.1
**SD**	0.6	1.2	0.5	1.5	0.8	2.4	0.9	2.9	0.8	3.7	0.6	1.1	0.8	1.3	0.9	2.6	1.0	5.9	1.0	10.2
**Median**	5.0	27.3	5.0	26.8	7.0	26.1	10.0	24.1	14.0	23.0	5.0	27.3	10.0	22.3	18.0	17.5	28.0	17.6	39.0	11.2
**Min**	4.0	22.2	4.0	21.5	5.0	19.1	7.0	18.2	12.0	14.0	4.0	22.2	8.0	15.9	15.0	9.0	24.0	0.0	36.0	0.0
**Max**	6.0	39.4	6.0	36.8	9.0	38.8	12.0	38.0	16.0	63.1	6.0	39.1	13.0	31.5	22.0	34.5	31.0	45.0	43.0	91.7
**Treatment intent**	**CURATIVE+PALLIATIVE**																			
***r***	**0.75**		**0.8**		**0.85**		**0.9**		**0.95**		**0.75**		**0.8**		**0.85**		**0.9**		**0.95**	
	**No**	**∆%**	**No**	**∆%**	**No**	**∆%**	**No**	**∆%**	**No**	**∆%**	**No**	**∆%**	**No**	**∆%**	**No**	**∆%**	**No**	**∆%**	**No**	**∆%**
**Mean**	10.6	34.4	12.8	34.4	16.5	34.8	25.8	33.1	39.0	30.8	10.6	34.5	33.8	43.7	52.1	57.7	65.4	61.4	86.6	61.0
**SD**	1.0	6.7	0.9	7.2	1.1	7.6	1.3	8.2	1.3	9.1	1.0	6.8	1.4	12.1	1.6	27.1	1.6	36.1	1.3	52.3
**Median**	11.0	32.0	13.0	31.7	17.0	32.3	26.0	30.8	39.0	27.9	11.0	32.1	34.0	41.6	52.0	55.9	65.0	50.6	87.0	38.2
**Min**	7.0	24.3	10.0	22.9	13.0	22.0	21.0	19.8	34.0	12.9	7.0	26.0	29.0	19.6	46.0	8.6	59.0	6.3	82.0	6.7
**Max**	14.0	70.6	16.0	66.7	20.0	69.2	30.0	64.9	43.0	122.8	14.0	70.3	40.0	100.0	60.0	156.2	71.0	196.9	91.0	252.3

*Abbreviations*: *A1* grouping strategy where all elements are required to correlate with one main element, *A2* grouping strategy where all elements are requeired to correlate with each other; *r* correlation coefficient

**Fig. 3:**
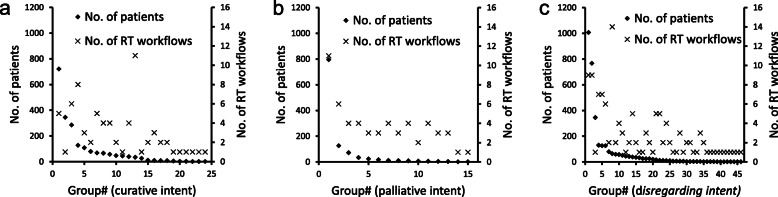
Total number of patients (left y-axis) for the groups aggregated together in A2_85 and the corresponding number of RT workflows per group (right y-axis) for curative treatment intent (**a**), pallative treatment intent (**b**) and disregareding treatment intent (**c**)

Using *A2*, where all elements in a RT workflow group were to correlate with each other, the different *r-value* cutoffs resulted in a minimum of 7/15/24/33/40 groups for curative intent, 4/8/15/24/36 groups for palliative intent, and 7/29/46/59/82 when treatment intent was disregarded (Table 4, right; Details for A2_85 in Fig. 3a-c). Corresponding absolute percentage differences for resource use between included elements were 109/160/190/258/279%, 39/32/35/45/92%, and 70/100/156/197/252%, respectively.

**Fig. 4:**
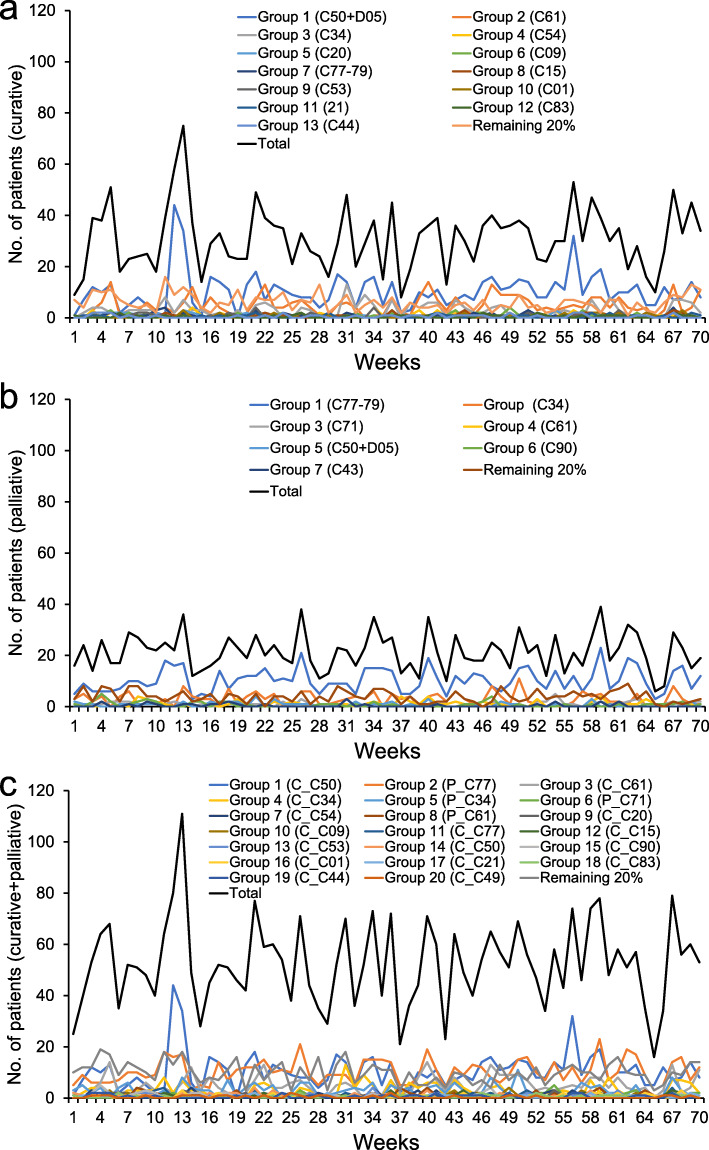
Inflow pattern of patients by intent: curative (**a**), palliative (**b**) and in total disregarding intent (**c**). Figure key shows Group# and corresponding ICD-10 code and for (**c**) the treatment intent is indicated by a C or P (curative or palliative respectively) before the ICD-10 code

Using the 80/20 Pareto rule resulted in 14 RT workflow groups for curative intent, 8 groups for palliative intent, and 21 groups when treatment intent was disregarded, with an inflow pattern as illustrated in Fig. 4a-c (curative, palliative and total). Since the number of groups by the customized algorithm at *r*=0.85 were comparable to or exceeded the number of groups by the 80/20 Pareto rule, modelling results were compared and reported for the two grouping strategies up to *r*=≤0.85 only. Number of patients waiting were subsequently investigated for nine RT workflow group scenarios including the reference which handled all RT workflows separately: *reference*, *Pareto_80/20, all-in-one, A1_75*, *A2_75*, *A1_80*, *A2_80*, *A1_85*, and *A2_85*.

### Modelling results

For *A1_75*, there were ≤28 RT workflows for each of the seven curative groups (1 single), ≤49 RT workflows for the four palliative groups (2 singles), and ≤80 RT workflows for the seven groups when treatment intent was disregarded (1 single). For *A1_80*, corresponding figures were ≤25, ≤49, and ≤71 (each with 2 singles) and for *A1_85*, there were ≤19, ≤39, and ≤54 (3, 2, and 1 single(s), respectively).

For *A2_75*, there were ≤31 RT workflows per each of the seven curative groups (1 single), ≤49 RT workflows for the four palliative groups (2 singles), and ≤72 RT workflows for the seven groups when treatment intent was disregarded (1 single). For *A2_80*, corresponding figures were ≤15, ≤32, and ≤19 (3, 3, and 12 singles, respectively) and for *A2_85*, there were ≤11, ≤11, and ≤14 (9, 2, and 21 singles, respectively).

### Performance of different grouping strategies

**Fig. 5:**
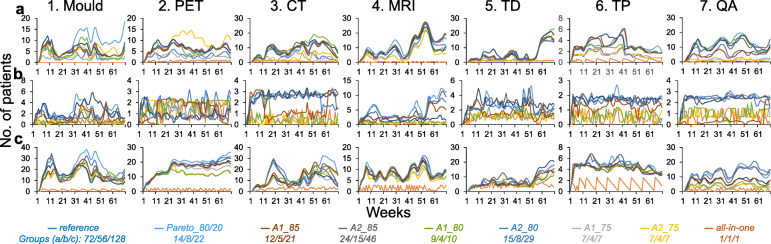
Number of patients waiting to various steps of the RT process by week as given by the output of the proposed simulation model over RT preparatory steps. RT tasks from left to right: 1. Mould, 2.PET, 3. CT, 4. MRI, 5. Target definition (TD), 6. Treatment planning (TP), and 7. QA. Top row corresponding to results for curative treatment intent (**a**), middle row for palliative treatment intent (**b**) and bottom row when disregarding treatment intent (**c**). The experiment includes nine different group scenarios: reference=each RT workflow handled separately, Pareto_80/20=80% of workflows handled separately and remaining 20% merged into one additional group, A1/2_75/80/85=proposed grouping strategies where all elements correlated with one main element [1]/correlated with each other [2] for utilization rate correlation coefficient cutoffs up to *r*=0.75/0.80/0.85, and all-in-one=all RT workflows merged into one group. Abbreviations: CT= Computed Tomography, MRI= Magnetic Resonance Imaging, PET= Positron Emission Tomography, QA=Quality Assurance, RT= Radiotherapy, TD=Target definition and TP=Treatment planning

Overall performance of the different grouping strategies compared with the reference are presented in Fig. 5 and Tables 5, 6 and 7.

**Table 5:** Absolute and relative differences for number of patients waiting between the investigated RT workflow grouping strategies and the reference scenario for curative treatment intent. Bold numbers indicate top three performing (lowest relative absolute mean) grouping strategies per task

	CURATIVE	***R***	***Pareto_80/20***	|∆|	|∆|/***R***	***A1_85***	|∆|	|∆|/***R***	***A2_85***	|∆|	|∆|/***R***	***A1_80***	|∆|	|∆|/***R***	***A2_80***	|∆|	|∆|/***R***	***A1_75***	|∆|	|∆|/***R***	***A2_75***	|∆|	|∆|/***R***	***all-in-one***	|∆|	|∆|/***R***
*1. Mould*	**mean**	9.03	2.86	6.16	65%	4.83	4.21	**40%**	5.07	4.01	**36%**	4.33	4.69	51%	5.34	4.01	**39%**	1.78	7.25	78%	4.41	4.69	49%	0.00	9.03	100%
	**sd**	4.14	1.68	3.70	19%	2.19	3.30	26%	2.21	3.52	29%	2.92	2.96	22%	2.69	3.04	24%	0.89	3.74	10%	2.08	3.24	20%	0.00	4.14	0%
	**median**	9.02	2.14	6.92	70%	4.33	5.59	52%	4.56	5.08	48%	3.16	4.24	45%	4.54	5.09	43%	1.55	7.64	79%	4.54	3.97	51%	0.00	9.02	100%
	**min**	0.00	0.00	0.00	20%	0.00	0.00	0%	0.00	0.00	0%	0.00	0.00	5%	0.00	0.00	0%	0.00	0.00	56%	0.00	0.00	1%	0.00	0.00	100%
	**max**	18.56	7.27	13.11	88%	10.47	9.88	71%	10.12	10.49	71%	11.54	12.88	83%	12.11	10.03	71%	5.20	15.64	92%	8.47	13.69	87%	0.00	18.56	100%
*2. PET*	**mean**	6.24	3.24	2.99	47%	6.07	0.52	**9%**	6.22	0.57	**10%**	5.77	0.92	16%	6.50	0.79	**13%**	2.79	3.44	52%	8.87	2.88	53%	0.61	5.62	89%
	**sd**	1.99	1.10	1.08	8%	1.55	0.40	7%	1.71	0.43	8%	2.15	0.79	12%	2.06	0.75	11%	0.98	1.80	18%	3.54	2.95	61%	0.32	1.96	6%
	**median**	6.19	3.05	3.05	48%	6.14	0.44	8%	6.19	0.54	8%	6.07	0.74	13%	6.51	0.48	10%	2.70	3.41	58%	8.44	1.32	18%	0.52	5.58	91%
	**min**	0.00	0.00	0.00	28%	0.00	0.00	0%	0.00	0.00	0%	0.00	0.00	0%	0.00	0.00	0%	0.00	0.00	8%	0.00	0.00	0%	0.00	0.00	73%
	**max**	10.33	5.91	4.69	63%	9.05	1.65	30%	9.04	1.40	34%	10.14	2.87	44%	10.42	2.69	41%	5.06	6.38	82%	14.51	9.80	229%	1.03	9.85	100%
*3. CT*	**mean**	7.94	4.77	3.41	42%	8.56	1.28	**21%**	8.48	1.09	**16%**	10.22	3.07	46%	9.00	2.03	**33%**	3.45	4.71	54%	3.14	4.79	61%	0.83	7.11	87%
	**sd**	3.79	2.94	2.93	23%	3.72	0.85	19%	3.84	1.43	22%	4.96	2.06	42%	4.17	1.39	31%	2.01	3.39	22%	2.23	2.51	15%	0.35	3.72	9%
	**median**	8.06	4.39	2.28	40%	8.61	1.22	15%	9.01	0.51	7%	9.78	3.01	32%	10.03	1.70	22%	3.07	4.25	56%	2.01	5.00	61%	1.01	7.29	89%
	**min**	0.00	0.00	0.00	1%	0.00	0.00	0%	0.00	0.00	0%	0.00	0.00	1%	0.00	0.00	1%	0.00	0.00	6%	0.00	0.00	30%	0.00	0.00	59%
	**max**	14.51	12.15	9.64	79%	14.77	3.26	78%	14.34	5.49	87%	19.09	6.59	212%	15.41	5.35	166%	8.48	11.45	88%	7.95	9.51	87%	1.44	13.49	100%
*4. MRI*	**mean**	12.02	8.65	3.37	35%	10.94	1.74	**19%**	10.93	1.58	**16%**	9.19	3.15	26%	9.50	2.75	**25%**	6.60	5.42	50%	6.07	5.95	53%	0.34	11.69	95%
	**sd**	6.38	6.64	1.78	21%	7.22	1.21	14%	6.88	1.09	12%	5.55	2.40	15%	6.00	2.06	15%	5.57	2.43	18%	5.42	2.71	18%	0.29	6.35	10%
	**median**	11.93	7.60	3.41	30%	8.71	1.49	21%	9.19	1.51	16%	7.66	2.09	23%	8.16	2.04	23%	3.96	5.23	50%	4.23	5.77	58%	0.26	11.60	97%
	**min**	0.00	0.00	0.00	3%	0.00	0.00	0%	0.00	0.00	0%	0.00	0.00	0%	0.00	0.00	0%	0.00	0.00	12%	0.00	0.00	5%	0.00	0.00	17%
	**max**	27.29	26.28	7.34	81%	27.62	5.18	58%	26.68	4.46	53%	21.58	8.51	63%	24.10	8.09	56%	22.43	12.03	86%	22.32	12.16	86%	0.89	26.91	100%
*5. TD*	**mean**	5.63	4.70	1.26	34%	5.50	0.63	**18%**	5.59	0.49	**11%**	6.02	1.36	**26%**	6.52	1.58	37%	3.99	1.73	42%	4.14	1.92	51%	1.10	4.53	70%
	**sd**	5.08	4.89	0.65	19%	5.14	0.47	15%	5.21	0.45	9%	6.52	1.58	21%	6.02	1.41	39%	4.99	1.27	23%	6.02	1.40	27%	0.41	5.02	19%
	**median**	3.23	2.24	1.29	39%	3.51	0.54	16%	3.41	0.36	10%	3.32	0.76	19%	4.40	1.10	23%	1.81	1.43	43%	1.13	1.69	66%	1.21	2.13	72%
	**min**	0.00	0.00	0.00	1%	0.00	0.00	0%	0.00	0.00	0%	0.00	0.00	1%	0.00	0.00	0%	0.00	0.00	3%	0.00	0.00	0%	0.00	0.00	28%
	**max**	17.90	15.13	3.15	61%	17.60	2.51	62%	17.75	1.97	35%	22.92	8.83	118%	20.94	5.46	172%	16.54	6.56	81%	18.78	6.70	80%	1.55	16.83	100%
*6. TP*	**mean**	2.90	1.46	1.44	49%	3.06	0.58	**21%**	3.06	0.53	**18%**	2.49	0.49	**15%**	3.10	0.67	24%	1.48	1.42	48%	1.53	1.37	47%	0.36	2.55	86%
	**sd**	0.83	0.58	0.56	15%	1.23	0.57	19%	1.25	0.58	19%	0.56	0.54	14%	1.29	0.62	21%	0.56	0.62	16%	0.59	0.56	15%	0.24	0.86	12%
	**median**	2.89	1.39	1.46	52%	2.91	0.43	16%	2.77	0.25	8%	2.66	0.27	10%	2.67	0.47	17%	1.42	1.37	51%	1.53	1.37	46%	0.38	2.47	88%
	**min**	0.00	0.00	0.00	6%	0.00	0.00	0%	0.00	0.00	0%	0.00	0.00	1%	0.00	0.00	0%	0.00	0.00	5%	0.00	0.00	2%	0.00	0.00	18%
	**max**	4.86	2.69	2.59	72%	6.16	3.21	109%	5.85	2.79	94%	3.28	2.33	65%	5.18	2.28	79%	2.73	2.86	73%	2.84	2.57	73%	0.78	4.84	100%
*7. QA*	**mean**	11.39	6.34	5.29	43%	5.10	6.29	53%	10.50	1.94	**17%**	11.01	1.59	**17%**	10.74	1.84	**19%**	2.01	9.39	79%	1.25	10.14	87%	0.36	11.03	97%
	**sd**	4.69	2.85	3.61	19%	2.12	3.81	18%	3.73	1.24	10%	4.34	1.34	17%	4.19	1.53	18%	1.13	4.63	16%	0.53	4.47	8%	0.25	4.63	2%
	**median**	11.31	6.07	5.12	44%	5.29	6.11	55%	10.10	1.71	17%	11.17	1.27	11%	10.99	1.55	14%	1.75	9.30	85%	1.36	9.72	89%	0.33	10.79	97%
	**min**	0.00	0.00	0.00	3%	0.00	0.00	3%	0.00	0.00	0%	0.00	0.00	0%	0.00	0.00	0%	0.00	0.00	24%	0.00	0.00	43%	0.00	0.00	89%
	**max**	19.65	12.72	11.85	75%	9.78	13.59	82%	16.06	4.63	47%	18.29	5.60	66%	18.17	6.71	89%	6.27	16.75	94%	2.44	17.82	96%	0.79	19.26	100%
**Total**	**mean**	7.88	4.57	3.42	45%	6.29	2.18	**26%**	7.12	1.46	**18%**	7.00	2.18	28%	7.24	1.95	**27%**	3.16	4.77	58%	4.20	4.54	57%	0.51	7.37	89%
	**sd**	3.84	2.95	2.05	18%	3.31	1.51	17%	3.55	1.25	16%	3.86	1.67	20%	3.77	1.54	23%	2.31	2.56	18%	2.91	2.55	24%	0.26	3.81	8%
	**median**	7.52	3.84	3.36	46%	5.64	2.26	26%	6.46	1.42	16%	6.26	1.77	22%	6.75	1.77	22%	2.32	4.66	60%	3.32	4.12	56%	0.53	6.98	91%
	**min**	0.00	0.00	0.00	9%	0.00	0.00	1%	0.00	0.00	0%	0.00	0.00	1%	0.00	0.00	0%	0.00	0.00	16%	0.00	0.00	12%	0.00	0.00	55%
	**max**	16.16	11.74	7.48	74%	13.64	5.61	70%	14.26	4.46	60%	15.26	6.80	93%	15.19	5.80	96%	9.53	10.24	85%	11.04	10.32	106%	0.93	15.68	100%

*Abbreviations*: *CT* computed tomography, *MRI* magnetic resonance imaging, *PET* positron-emission tomography, *QA* quality assurance, *R* reference, *SD* standard deviation, *TD* target definition, and *TP* treatment planning, *│∆│* absolute difference, *│∆│/R* relative difference; notation of RT workflow grouping scenarios as described in Fig. 5

**Table 6:** Absolute and relative patient differences for number of patients waiting between the investigated RT workflow grouping strategies and the reference scenario for palliative treatment intent. Bold numbers indicate top three performing (lowest relative absolute mean) grouping strategies per task

	PALLIATIVE	***R***	***Pareto_80/20***	|∆|	|∆|/***R***	***A1_85***	|∆|	|∆|/***R***	***A2_85***	|∆|	|∆|/***R***	***A1_80***	|∆|	|∆|/***R***	***A2_80***	|∆|	|∆|/***R***	***A1_75***	|∆|	|∆|/***R***	***A2_75***	|∆|	|∆|/***R***	***all-in-one***	|∆|	|∆|/***R***
***1. Mould***	**mean**	2.47	1.69	0.86	**28%**	1.17	1.33	56%	1.53	0.97	**33%**	0.57	1.90	78%	2.51	0.85	**42%**	0.65	1.82	73%	0.64	1.83	75%	0.53	1.94	73%
	**sd**	1.21	0.73	1.07	23%	0.99	0.96	25%	0.70	1.01	21%	0.61	1.03	17%	1.37	0.87	58%	0.78	1.10	23%	0.78	1.07	22%	0.34	1.27	23%
	**median**	2.25	1.49	0.33	22%	0.73	1.08	62%	1.27	0.59	30%	0.40	1.65	79%	2.21	0.60	23%	0.45	1.65	79%	0.45	1.65	79%	0.50	1.78	79%
	**min**	0.00	0.00	0.00	1%	0.00	0.00	2%	0.00	0.00	1%	0.00	0.00	36%	0.00	0.00	0%	0.00	0.00	22%	0.00	0.00	22%	0.00	0.00	20%
	**max**	5.58	4.19	4.09	76%	3.91	4.97	96%	4.16	4.55	82%	2.76	5.26	99%	5.88	3.33	251%	3.38	5.27	100%	3.38	5.19	100%	1.09	5.50	100%
***2. PET***	**mean**	1.35	1.95	0.62	**60%**	1.41	0.91	84%	0.72	0.65	**42%**	1.73	0.89	92%	0.93	0.55	**39%**	1.85	0.80	89%	1.87	0.81	90%	1.38	0.72	63%
	**sd**	0.60	0.64	0.40	52%	0.91	0.56	67%	0.33	0.53	22%	0.66	0.54	83%	0.44	0.46	24%	0.54	0.53	97%	0.50	0.50	93%	0.66	0.57	58%
	**median**	1.33	1.96	0.53	38%	1.87	0.82	75%	0.66	0.54	41%	2.06	0.87	69%	0.83	0.46	40%	2.08	0.74	52%	2.07	0.80	52%	1.62	0.75	53%
	**min**	0.00	0.00	0.00	5%	0.00	0.00	0%	0.00	0.00	1%	0.00	0.00	0%	0.00	0.00	0%	0.00	0.00	0%	0.00	0.00	1%	0.00	0.00	0%
	**max**	2.94	3.19	1.75	234%	2.34	2.67	280%	1.99	2.30	89%	2.21	2.39	346%	2.54	2.04	105%	2.21	1.85	393%	2.21	1.68	374%	2.18	2.73	267%
***3. CT***	**mean**	2.71	2.25	0.48	**18%**	1.50	1.40	52%	2.53	0.31	**13%**	0.53	2.19	81%	2.60	0.17	**8%**	0.32	2.39	87%	0.29	2.41	88%	0.46	2.25	82%
	**sd**	0.54	0.56	0.40	14%	1.00	0.80	28%	0.59	0.31	16%	0.54	0.70	19%	0.54	0.14	11%	0.32	0.60	14%	0.29	0.59	13%	0.46	0.68	18%
	**median**	2.81	2.45	0.32	12%	1.10	1.70	62%	2.72	0.21	8%	0.43	2.40	84%	2.67	0.13	5%	0.22	2.55	91%	0.20	2.56	92%	0.30	2.43	89%
	**min**	0.00	0.00	0.00	0%	0.00	0.00	0%	0.00	0.00	0%	0.00	0.00	6%	0.00	0.00	0%	0.00	0.00	18%	0.00	0.00	18%	0.00	0.00	17%
	**max**	3.35	3.07	1.49	58%	3.21	3.00	131%	2.98	1.39	96%	2.96	3.03	100%	3.35	0.76	64%	1.31	3.32	100%	0.99	3.32	100%	1.45	3.29	100%
***4. MRI***	**mean**	5.14	4.44	0.91	**17%**	2.96	2.31	**39%**	3.57	1.59	**38%**	0.70	4.46	81%	1.85	3.31	53%	0.71	4.44	79%	0.72	4.43	79%	0.41	4.74	89%
	**sd**	3.22	2.41	1.00	12%	1.79	2.04	24%	3.00	0.78	17%	0.46	3.17	17%	0.56	3.00	25%	0.42	3.27	19%	0.40	3.26	19%	0.38	3.22	14%
	**median**	4.05	3.49	0.55	14%	2.36	1.62	38%	2.35	1.56	38%	0.67	3.63	87%	1.87	2.19	59%	0.66	3.30	86%	0.69	3.31	86%	0.22	3.70	94%
	**min**	0.00	0.00	0.00	0%	0.00	0.00	1%	0.00	0.00	5%	0.00	0.00	31%	0.00	0.00	0%	0.00	0.00	14%	0.00	0.00	14%	0.00	0.00	23%
	**max**	12.32	9.60	3.58	47%	7.67	6.30	89%	11.36	3.95	76%	1.79	11.72	99%	3.22	10.53	86%	1.56	11.71	100%	1.56	11.66	100%	1.45	12.24	100%
***5. TD***	**mean**	2.46	2.32	0.36	**14%**	1.63	1.06	43%	2.48	0.45	**18%**	1.28	1.21	46%	2.76	0.71	**31%**	1.16	1.31	49%	1.17	1.30	49%	0.92	1.54	60%
	**sd**	0.92	0.77	0.42	13%	0.83	0.77	21%	0.94	0.41	13%	0.49	0.84	21%	0.73	0.60	27%	0.45	0.94	22%	0.46	0.94	22%	0.44	0.87	21%
	**median**	2.24	2.24	0.22	11%	1.44	0.88	42%	2.59	0.43	17%	1.31	1.06	50%	2.73	0.60	23%	1.18	1.11	53%	1.20	1.11	53%	0.92	1.47	62%
	**min**	0.00	0.00	0.00	0%	0.00	0.00	0%	0.00	0.00	1%	0.00	0.00	0%	0.00	0.00	1%	0.00	0.00	6%	0.00	0.00	3%	0.00	0.00	1%
	**max**	5.32	4.89	2.37	86%	3.46	4.18	120%	4.96	2.47	53%	2.71	3.95	85%	4.95	2.81	131%	2.19	4.58	86%	2.44	4.52	86%	1.73	4.23	99%
***6. TP***	**mean**	1.70	1.71	0.19	**11%**	1.20	0.51	30%	1.60	0.20	**11%**	0.57	1.12	66%	1.71	0.22	**13%**	0.56	1.14	67%	0.59	1.11	65%	0.46	1.23	73%
	**sd**	0.35	0.28	0.15	8%	0.32	0.28	15%	0.28	0.20	9%	0.33	0.41	20%	0.28	0.18	11%	0.33	0.42	20%	0.32	0.39	19%	0.36	0.43	22%
	**median**	1.72	1.74	0.15	9%	1.27	0.50	28%	1.60	0.15	9%	0.55	1.11	65%	1.77	0.16	9%	0.54	1.16	68%	0.60	1.15	63%	0.35	1.32	81%
	**min**	0.00	0.00	0.00	1%	0.00	0.00	0%	0.00	0.00	0%	0.00	0.00	5%	0.00	0.00	0%	0.00	0.00	19%	0.00	0.00	31%	0.00	0.00	17%
	**max**	2.73	2.16	0.81	32%	1.81	1.33	71%	2.09	1.17	43%	1.19	2.01	100%	2.07	0.86	46%	1.19	1.81	100%	1.13	1.81	99%	1.13	1.98	99%
***7. QA***	**mean**	2.44	2.28	0.28	**12%**	0.37	2.08	85%	2.33	0.18	**8%**	1.07	1.38	56%	2.24	0.25	**11%**	0.86	1.59	68%	0.84	1.61	69%	0.83	1.63	71%
	**sd**	0.48	0.51	0.24	12%	0.53	0.67	21%	0.56	0.22	12%	0.49	0.60	20%	0.47	0.16	10%	0.40	0.49	28%	0.41	0.49	28%	0.48	0.53	38%
	**median**	2.52	2.44	0.20	8%	0.27	2.30	90%	2.42	0.11	4%	1.30	1.19	49%	2.38	0.24	10%	0.75	1.70	70%	0.74	1.71	70%	0.75	1.67	70%
	**min**	0.00	0.00	0.00	0%	0.00	0.00	8%	0.00	0.00	0%	0.00	0.00	7%	0.00	0.00	0%	0.00	0.00	37%	0.00	0.00	37%	0.00	0.00	35%
	**Max**	3.10	2.68	1.01	69%	2.30	2.81	100%	2.91	1.08	65%	2.04	2.98	96%	2.63	0.67	76%	1.51	3.03	261%	1.51	3.04	261%	1.51	2.64	348%
**Total**	**Mean**	2.61	2.38	0.53	**23%**	1.46	1.37	56%	2.11	0.62	**23%**	0.92	1.88	71%	2.09	0.87	**28%**	0.87	1.93	73%	0.87	1.93	73%	0.71	2.01	73%
	**Sd**	1.05	0.84	0.53	19%	0.91	0.87	29%	0.91	0.49	16%	0.51	1.04	28%	0.63	0.77	24%	0.46	1.05	32%	0.45	1.03	31%	0.45	1.08	28%
	**Median**	2.42	2.26	0.33	16%	1.29	1.27	57%	1.94	0.51	21%	0.96	1.70	69%	2.07	0.62	24%	0.84	1.75	71%	0.85	1.76	71%	0.67	1.88	75%
	**Min**	0.00	0.00	0.00	1%	0.00	0.00	2%	0.00	0.00	1%	0.00	0.00	12%	0.00	0.00	0%	0.00	0.00	16%	0.00	0.00	18%	0.00	0.00	16%
	**Max**	5.05	4.25	2.16	86%	3.53	3.61	127%	4.35	2.41	72%	2.24	4.48	132%	3.52	3.00	108%	1.91	4.51	163%	1.89	4.46	160%	1.51	4.66	159%

*Abbreviations*: *CT* computed tomography, *MRI* magnetic resonance imaging, *PET* positron-emission tomography, *QA* quality assurance, *R* reference, *SD* standard deviation, *TD* target definition, and *TP* treatment planning, *│∆│* absolute difference, *│∆│/R* relative difference; notation of RT workflow grouping scenarios as described in Fig. 5

**Table 7:** Absolute and relative differences for number of patients waiting between the investigated workflow grouping strategies and the reference scenario disregarding treatment intent. Bold numbers indicate top three performing (lowest relative absolute mean) grouping strategies per task

	CURATIVE+ PALLIATIVE	***R***	***Pareto_80/20***	|∆|	|∆|/***R***	***A1_85***	|∆|	|∆|/***R***	***A2_85***	|∆|	|∆|/***R***	***A1_80***	|∆|	|∆|/***R***	***A2_80***	|∆|	|∆|/***R***	***A1_75***	|∆|	|∆|/***R***	***A2_75***	|∆|	|∆|/***R***	***all-in-one***	|∆|	|∆|/***R***
***1. Mould***	**mean**	20.62	18.57	4.41	**22%**	14.93	5.80	**26%**	16.27	5.50	**25%**	14.20	7.02	30%	14.29	7.29	33%	12.72	7.91	35%	14.22	6.80	30%	1.36	19.27	93%
	**sd**	9.49	7.57	2.91	14%	6.40	4.91	16%	7.25	4.16	12%	4.86	6.24	19%	6.43	6.64	20%	4.91	6.16	18%	5.12	5.50	17%	0.81	9.38	6%
	**median**	18.88	16.93	3.57	18%	13.31	3.88	21%	15.23	4.06	25%	13.58	3.57	26%	14.42	4.77	34%	12.40	6.36	35%	13.51	5.73	33%	1.38	17.52	94%
	**min**	0.00	0.00	0.00	2%	0.00	0.00	1%	0.00	0.00	0%	0.00	0.00	1%	0.00	0.00	1%	0.00	0.00	1%	0.00	0.00	3%	0.00	0.00	74%
	**max**	37.97	33.13	10.84	69%	29.39	17.58	81%	34.44	13.62	64%	23.48	18.87	86%	25.52	23.30	72%	24.06	22.64	83%	24.91	20.48	87%	2.61	36.74	100%
***2. PET***	**mean**	18.08	16.57	1.51	**9%**	15.55	2.57	13%	16.08	2.04	**12%**	11.39	6.73	34%	15.70	2.42	**12%**	14.08	4.02	20%	11.66	6.44	32%	1.22	16.88	92%
	**sd**	5.84	5.52	0.69	4%	4.37	1.78	7%	5.14	0.93	5%	2.65	3.88	14%	4.53	1.48	5%	3.83	2.39	8%	2.86	3.82	14%	0.87	5.91	11%
	**median**	20.23	18.56	1.50	8%	17.48	2.45	13%	17.82	1.93	11%	11.57	8.18	42%	17.49	2.50	12%	15.46	4.00	21%	11.92	7.63	38%	1.14	19.01	94%
	**min**	0.00	0.00	0.00	3%	0.00	0.00	1%	0.00	0.00	2%	0.00	0.00	3%	0.00	0.00	0%	0.00	0.00	1%	0.00	0.00	2%	0.00	0.00	34%
	**max**	26.82	23.59	3.30	23%	20.35	8.06	30%	23.06	4.41	29%	15.70	14.44	54%	21.25	6.52	26%	18.23	10.15	38%	17.59	13.59	51%	3.59	26.14	100%
***3. CT***	**mean**	15.30	12.77	4.71	**28%**	13.16	4.61	**32%**	12.68	3.49	**20%**	12.54	4.89	32%	9.71	6.67	37%	8.52	7.40	40%	11.34	5.29	32%	2.49	12.80	79%
	**sd**	9.38	7.18	4.83	18%	7.52	3.27	21%	7.45	3.71	16%	6.07	4.15	19%	4.80	6.05	20%	3.93	6.60	21%	5.22	4.77	18%	1.11	9.00	16%
	**median**	14.25	11.82	2.93	25%	12.83	4.70	30%	11.21	2.00	20%	12.26	3.83	32%	9.02	5.07	40%	7.46	6.33	42%	10.41	3.48	32%	2.46	11.04	84%
	**min**	0.00	0.00	0.00	3%	0.00	0.00	1%	0.00	0.00	1%	0.00	0.00	2%	0.00	0.00	4%	0.00	0.00	3%	0.00	0.00	2%	0.00	0.00	29%
	**max**	32.33	29.87	16.55	75%	26.93	10.23	118%	28.33	13.83	69%	23.76	15.68	78%	18.60	19.20	73%	16.47	21.15	81%	19.99	16.60	85%	4.76	30.52	100%
***4. MRI***	**mean**	9.04	8.39	1.14	13%	8.41	0.80	**8%**	8.37	0.81	**9%**	7.36	1.70	18%	7.90	1.18	**13%**	6.51	2.54	26%	6.19	2.87	30%	1.48	7.56	82%
	**sd**	2.91	3.36	0.85	9%	2.50	0.71	6%	2.89	0.80	8%	2.29	0.96	8%	2.48	0.86	8%	1.76	1.45	10%	1.76	1.61	12%	1.09	2.93	16%
	**median**	8.88	8.03	0.88	11%	8.10	0.70	8%	8.11	0.44	6%	7.39	1.57	18%	7.60	0.89	13%	6.09	2.50	26%	5.77	2.52	31%	1.44	7.25	86%
	**min**	0.00	0.00	0.00	0%	0.00	0.00	0%	0.00	0.00	0%	0.00	0.00	1%	0.00	0.00	1%	0.00	0.00	5%	0.00	0.00	4%	0.00	0.00	8%
	**max**	14.75	16.26	2.82	38%	13.56	2.73	26%	15.33	3.34	36%	11.83	3.76	33%	14.16	3.35	30%	10.85	5.40	48%	10.02	5.57	49%	3.06	13.97	100%
***5. TD***	**mean**	6.92	7.63	1.45	24%	6.47	0.94	**17%**	7.69	2.43	40%	5.15	1.97	26%	7.77	1.58	22%	6.74	0.85	**16%**	5.64	1.58	**21%**	3.96	3.20	39%
	**sd**	4.06	3.61	1.24	23%	3.88	0.83	15%	3.17	1.80	31%	2.34	2.03	16%	5.59	1.35	14%	4.43	0.59	15%	2.80	1.67	15%	1.49	3.73	25%
	**median**	5.40	6.98	1.00	16%	4.70	0.74	14%	8.07	2.12	30%	4.57	1.21	24%	5.46	1.23	23%	5.05	0.80	12%	4.84	1.02	20%	4.12	2.01	41%
	**min**	0.00	0.00	0.00	0%	0.00	0.00	0%	0.00	0.00	0%	0.00	0.00	1%	0.00	0.00	0%	0.00	0.00	0%	0.00	0.00	0%	0.00	0.00	1%
	**max**	18.45	14.37	4.72	92%	15.62	2.92	82%	14.50	6.74	114%	11.48	7.17	88%	21.04	4.63	56%	17.37	2.57	81%	13.38	6.66	82%	6.19	15.62	100%
***6. TP***	**mean**	4.81	4.57	0.45	**9%**	4.09	0.76	15%	4.68	0.49	**10%**	4.23	0.64	**12%**	4.20	0.72	15%	4.08	0.74	15%	4.20	0.69	13%	1.35	3.47	70%
	**sd**	1.06	0.92	0.44	7%	0.81	0.55	9%	1.17	0.46	9%	0.78	0.63	10%	0.94	0.54	10%	0.84	0.58	10%	0.77	0.61	10%	0.77	1.29	19%
	**median**	4.89	4.61	0.31	7%	4.37	0.58	12%	4.76	0.29	7%	4.49	0.37	9%	4.47	0.58	12%	4.37	0.55	12%	4.42	0.50	12%	1.35	3.49	72%
	**min**	0.00	0.00	0.00	0%	0.00	0.00	2%	0.00	0.00	0%	0.00	0.00	0%	0.00	0.00	1%	0.00	0.00	1%	0.00	0.00	0%	0.00	0.00	14%
	**max**	6.90	6.64	2.13	33%	5.09	2.49	36%	6.86	1.63	38%	5.07	2.45	38%	5.55	2.31	39%	4.85	2.33	46%	5.05	2.53	39%	2.61	6.15	99%
***7. QA***	**mean**	12.30	12.87	1.55	**14%**	6.54	5.77	47%	11.39	1.47	**15%**	4.20	8.11	66%	7.18	5.12	**40%**	3.08	9.22	75%	3.55	8.76	71%	1.18	11.13	90%
	**sd**	4.14	4.24	1.28	13%	2.26	2.67	13%	4.28	0.95	12%	1.50	3.07	8%	2.11	2.53	11%	1.25	3.28	6%	1.32	3.25	7%	0.87	4.07	8%
	**median**	12.14	13.44	1.23	11%	6.90	5.79	50%	11.61	1.54	14%	4.33	8.25	66%	7.65	4.85	44%	2.90	9.36	75%	3.44	8.76	72%	1.07	11.17	90%
	**min**	0.00	0.00	0.00	0%	0.00	0.00	16%	0.00	0.00	0%	0.00	0.00	40%	0.00	0.00	13%	0.00	0.00	61%	0.00	0.00	57%	0.00	0.00	67%
	**max**	19.11	20.59	4.66	74%	10.23	10.48	81%	16.93	3.63	68%	6.73	12.93	87%	10.34	10.16	56%	5.40	14.60	87%	5.64	14.37	87%	2.78	18.19	100%
**Total**	**mean**	12.44	11.63	2.17	**17%**	9.88	3.04	**22%**	11.02	2.32	**19%**	8.44	4.44	31%	9.53	3.57	25%	7.96	4.67	32%	8.11	4.63	33%	1.86	10.61	78%
	**sd**	5.27	4.63	1.75	13%	3.96	2.10	12%	4.48	1.83	13%	2.93	2.99	13%	3.84	2.78	13%	2.99	3.01	13%	2.84	3.03	13%	1.00	5.19	14%
	**median**	12.09	11.48	1.63	14%	9.67	2.69	21%	10.97	1.77	16%	8.31	3.85	31%	9.44	2.84	25%	7.68	4.27	32%	7.76	4.23	34%	1.85	10.21	80%
	**min**	0.00	0.00	0.00	1%	0.00	0.00	3%	0.00	0.00	1%	0.00	0.00	7%	0.00	0.00	3%	0.00	0.00	10%	0.00	0.00	10%	0.00	0.00	32%
	**max**	22.33	20.64	6.43	58%	17.31	7.78	65%	19.92	6.74	60%	14.01	10.76	66%	16.64	9.93	50%	13.89	11.26	66%	13.80	11.40	69%	3.66	21.05	100%

*Abbreviations*: *CT* computed tomography, *MRI* magnetic resonance imaging, *PET* positron-emission tomography, *QA* quality assurance, *R* reference, *SD* standard deviation, *TD* target definition, and *TP* treatment planning, *│∆│* absolute difference, *│∆│/R* relative difference; notation of RT workflow grouping scenarios as described in Fig. 5

When assessing the mean absolute differences in number of patient waiting between all grouping strategies compared with the reference, *all-in-one* consistently performed worst and *A2_85* generally performed best (curative/palliative/disregarding treatment intent: 7.4±3.8/2.0±1.1/10.6±5.2 patients versus 1.5±1.2/0.6±0.5/2.3±1.8 patients; Fig. 5; Tables 5, 6 and 7). Performance of *Pareto_80/20* were generally second best to *A2_85* (3.4±2.0/0.5±0.5/2.1±1.7 patients). For the same *r*-value cutoff, results by grouping strategy *A2* typically performed better than results by grouping strategy *A1*.

When assessing the impact of the grouping strategies on mean relative differences in number of patients waiting for each RT preparatory task, the smallest differences (least dependent on grouping strategy) were generally found for *5. Target* definition and the largest (most dependent on grouping strategy) for *7. QA* (Fig. 5; Tables 5, 6 and 7). However, patterns between treatment intents varied and the overall smallest differences were found for all tasks except for *1. Mould* and correspondingly for overall largest differences except for *6. Treatment planning*.

Assessing the performance of the grouping strategy based on median values instead of mean values were also investigated, with comparable results (data not shown).

## Discussion

In this work, we investigated the performance of various strategies to group care pathways for patients with different cancer diagnoses and treatment intents referred to RT (RT workflows). Our aim was to identify an aggregated input data set to a sub-structure of a SD model capturing the preparatory steps of the RT process, which produced similar results as the original patient referral pattern. Using data from 3209 patients treated in 2015-2016 through 128 care pathways at a modern seven-linac RT department, we found that the accepted 80/20 Pareto rule performed well with respect to number of patients waiting, but that even better results could be achieved if using a grouping strategy, which recognized similarities in resource use. The latter, however, resulted in a somewhat more extensive input data set compared with the former.

Needing to reduce dimensions of input data prior to modelling is a common problem when working with real world data [13]. A well-designed dimension reduction strategy creates a smaller input data set, which provides the same modelling results as the original representation. To this end, some measurement of similarity is important to use and for the problem addressed in this work, there is to our knowledge no recommended way of deciding this (a PubMed search on March 21^st^, 2019, gave zero relevant hits for various combinations of “radiation therapy”, “simulation model”, “input data set”, “operations research”). Neither of the accepted strategies to handle input data of a simulation model, the Pareto rule nor the merging of all data into one group, use a similarity measure when reducing the input data structure. We, therefore, proposed to use pairwise correlations between groups based on RT preparatory step resource use for a number of reasons. As patients pass through the different steps of the RT process, available resources set the limit for to which extent the demand can be met. If the treatment of multiple cancer diagnosis requires a similar amount of resources, scheduling them for RT can be simplified by grouping them together. Conditions for RT also change over time, with emerging data for instance motivating fewer treatments for prostate cancer [14]. This is a large group of patients referred to RT and a similarity measure based on resource utilization rates can assist in understanding to which extent existing treatments already follow this pattern. If so, such a similarity measure can guide whether a new patient care pathway needs to be created or if those for another cancer diagnose or treatment intent can be reused. Finally, the underlying data for our proposed similarity measure can easily be extracted from existing oncological information systems and the measure in itself is straightforward to calculate. Although the purpose of our system dynamics model over the RT process is not to find an optimal solution to the problem at hand, it is interesting to note that none of the aforementioned OR models for RT by Vieira [7], Kapamara [9] and Proctor [10] discuss grouping strategies or similarity measures when quantifying patient volumes as either an alternative to or together with probability distributions as estimated from observed data.

The level of data aggregation needed to reduce model complexity will depend on the amount of details the model is intended to capture. System dynamics is an OR method that can help to understand the behaviors of a complex system in various scenarios [15]. Systems can be complex based on several reasons, for instance non-linear relations between cause and effect. There is a difference between complicated and complex systems, where a complicated system can be hard to understand while a complex system can consist of many easy steps influencing each other in a non-predictable, and even in a counterintuitive way. For the understanding of system behaviors after grouping data in the investigated RT scenario, a high level of aggregation will probably work to capture overall effects during long time periods. However, the total capacity needed to handle groups of patients requiring different resources or resource use can be different from the average level. With too high a level of aggregation, details get averaged out and when investigating effects for certain groups the result may not be noticeable. Different inflow patterns will also have an impact in this context as shown by our data. Fluctuating incoming flows is common in RT [7] and a steady state typically never exist. This needs to be kept in mind when considering how to aggregate data for situations where incoming flows are stable over time since the impact of grouping strategy may be of less importance. For a general setting in line with RT, where there might be variable incoming flows of objects instead of patients and those objects go through a multiple-step process with different requirements of resource use at each step, it is likely that the choice of aggregation method will have an effect on the end results as will the investigated period of time. Which aggregation method that is most appropriate needs, however, to be determined for that specific situation based on the characteristics of available data.

Strengths of this study include the use of real-world data from a large modern RT department, which captured yearly variations in the referral pattern of patients to RT, and an objective approach to evaluate accepted and new grouping strategies as a means to reduce the input data set. By including all patients with an ICD-code registered in ARIA®, and who were planned for RT during the investigated period, the effect of rarely treated diagnoses could be acknowledged for various grouping strategies. We handled the data in weekly batches in our model, although other studies have identified daily variations in referral patterns [7]. Potential effects by daily variations will be further investigated in a full-scale SD model instead of the current model which was primarily designed to illustrate effects of grouping strategies in a generic simulation modelling context. A future refined version of the simulation model will acknowledge feedback loops to allow for patients to reverse in the process with various tasks needing to be redone rather than the single-direction of patient flows which is supported by the current version. The current design may also explain why we found the largest dependence on grouping strategy in the last step of the RT preparatory tasks (QA), where number of patients waiting can be expected to accumulate by the chosen design. With the over 100 patient care pathways identified in this study, conditions for model building were excellent and effects by different grouping strategies could really be tested. However, investigated correlations were strong leading to a certain redundancy in using the Mann-Whitney statistic to guide in similarity as a pre-processing step for our customized grouping algorithm. This step would be more important for less strongly correlating data than for the information we explored here. We selected the smallest number of groups by each scenario for analysis without further evaluation of the included diagnoses or the within-group differences. If these or other criteria had been used for selection, larger groups would have been the consequence. Since we wanted to identify as few groups as possible, detailed investigation of other selection criteria was outside the scope of this study. Furthermore, results by the grouping strategies of our customized algorithm depended on the ordering of data and a permutation strategy was introduced to minimize these effects. The total number of ways to order a set of 100 or more elements is far bigger than what is possible to investigate exhaustively and we, therefore, decided on the proposed strategy with 10000 randomly selected orderings. Increasing the number to 100000 orderings had no impact on the reported results, however, reducing the number to 1000 failed to identify some of the smaller solutions (data not shown). Finally, the results presented here are specific for the investigated RT department. Produced results will always mirror the character of the incoming flow of patients during the studied period of time. The proposed aggregation strategy, however, can be expected to work with a different dataset in any general setting where resource usage can be used to quantify the similarity of a process.

## Conclusions

The need to group incoming patient flows to a simulation model over the RT process is important to maintain simplicity whilst acknowledging the highly variable data. The accepted 80/20 Pareto rule can be expected to perform well as a grouping strategy for this purpose but even better results may be achieved if using a grouping strategy where a similarity measure between RT care pathways are used to identify groups. Even if the latter requires pre-processing of data, finding similarities between groups, with or without consideration of treatment intents, it may also prove to be clinically useful in other situations. Such examples include capacity planning of RT and dynamic re-planning of different RT booking scenarios where reducing number of alternatives may be of importance whilst still needing to acknowledge the autonomy of each patient care pathway.

## Supplementary Information

**Additional file 1.** Grouping algorithm and simulation model description.

## Data Availability

Additional datasets used and/or analyzed during the current study are available from the corresponding author on reasonable request.

## Abbreviations

CT: Computed tomography
MRI: Magnetic resonance imaging
OR: Operations research
PET: Positron-emission tomography
QA: Quality assurance
RT: Radiotherapy
SD: System dynamics
TD: Target definition
TP: Treatment planning
