# Supplementary material for: An analytical approach to aggregate patient inflows to a simulation model over the radiotherapy process
Source: BMC Health Serv Res. 2021 Mar 8;21:207. doi: 10.1186/s12913-021-06162-4 (PMC7938525; doi:10.1186/s12913-021-06162-4)
Supplement: Supplementary file 1 — Additional file 1. Grouping algorithm and simulation model description. [file 12913_2021_6162_MOESM1_ESM.pdf]

Additional file 1 - Supplementary materials

Grouping algorithm

<u>Input:</u> data matrix (***rows***=workflows; ***columns***=RT preparatory tasks), correlation coefficient termination criterion ***r*** to decide degree of similarity with respect to resource use (***co***), and GroupingStrategy ***v*** (A1 or A2 as described in the article)
<u>Output:</u> workflow groups according to GroupingStrategy 1 or 2
compute all pairwise correlations between ***rows***; keep statistically significant correlations
repeat
select the first element of the randomly permutated ordering of ungrouped workflows [*current*]
while *current* correlates with another ungrouped workflow (mutual ***r***≥***co***)
do group the next ungrouped workflow with *current* according to
case(***v***)
v=1=> GroupingStrategy 1
v=2=> GroupingStrategy 2
end case
end do
select the next ungrouped workflow to be *current*
end while
until all workflows have been assigned to a group of similar resource use as defined by ***co***
return workflow groups

Simulation model description

Overall structure

The process of preparing a patient for radiation treatment consists of seven main operative steps. In the model, these are proceeded by two steps, ‘referral’ and ‘initiation’, and succeeded by one step, ‘to treatment’.
The seven main operative steps are:
1. *Positioning aid (Mould)*
2. *Positron-emission tomography (PET)*
3. *Computed tomography (CT)*
4. *Magnetic resonance imaging (MRI)*
5. *Target definition (TD)*
6. *Treatment planning (TP)*
7. *Patient quality assurance (QA)*

Each patient case is treated as an object that flows through the different operative steps in sequence. The flow through each step is determined by available capacity and also depends on the number of patients in waiting for the specific step. Patient volumes and flows vary over time with each diagnose and treatment intent requiring step-specific capacities.
The model handles this variation for different types of diagnoses with unique volumes, time variability and capacity requirement as specified by the input data (Supplementary table 1).

Supplementary table 1. Input data example for nine groups of diagnose specific capacity requirements, in percent.

Group	PPRCT Mould	PPRCT PET	PPRCT CT	PPRCT MRI	PPRCT Target definition	PPRCT Treatment planning	PPRCT QA
Group 1	11.72	1.56	13.28	18.75	100.00	128.13	60.16
Group 2	76.72	2.24	107.46	23.43	100.00	117.91	52.54
Group 3	18.51	0.00	113.10	0.63	100.00	117.38	1.51
Group 4	36.36	0.00	36.36	90.91	100.00	109.09	9.09
Group 5	375.00	4.17	416.67	33.33	100.00	112.50	50.00
Group 6	33.33	0.00	33.33	0.00	100.00	0.00	0.00
Group 7	6.11	52.67	73.28	4.58	100.00	124.43	89.31
Group 8	28.57	0.00	28.57	100.00	100.00	100.00	100.00
Group 9	0.29	0.00	97.98	97.98	100.00	102.89	68.79

Abbreviations: PPRCT=Percentage patients requiring capacity, CT=Computed Tomography, MRI=Magnetic Resonance Imaging, PET=Positron Emission Tomography, QA=Quality Assurance, and RT=Radiotherapy.

Model documentation

INFLOW STATS: Patient volumes over time (weekly batches) per diagnosis (ICD-code) and treatment intent (curative or palliative), 70 weeks 2015-2016
INFLOW: When the model is run, a flow of patients enters from imported statistics in INFLOW STATS into INFLOW with weekly distribution split into diagnosis groups (DG) from an input data spreadsheet.
Wait referral to RT: The accumulated number of patients waiting for referral. Calculated from the equation: Wait_referral_to_RT[DG_prep](t)=Wait_referral_to_RT[DG_prep](t - dt) + (INFLOW[DG_prep] - Referral_dispatch[DG_prep]) * dt
Where Where DG_prep is a defined structure (array) enabling parallel calculation with unique data for each diagnose group, t=time, dt=delta time (amount of time between calculations in the model).

Referral dispatch: Enables the flow of patients through the referral dispatch. Calculated from the equation: Physician_capacity*Wait_referral_to_RT
Physician capacity: A selected constant value (set to 1) chosen to allow for a steady inflow of patients.
Wait Initiation: Accumulation of number of patients waiting for initiation. Calculated from the equation: Wait_Initiation[DG_prep](t) = Wait_Initiation[DG_prep](t - dt) + (Referral_dispatch[DG_prep] - Initiation_of_RT_preparation[DG_prep]) * dt
Where Where DG_prep is a defined structure (array) enabling parallel calculation with unique data for each diagnose group, t=time, dt=delta time (amount of time between calculations in the model).

Initiation of RT preparation: Enables the flow of patients through the initiation dispatch. Calculated from the equation: Initiation_pace*Share_of_DG_Wait_start
Initiation pace: A selected constant value chosen to allow for a steady throughput of patients. This is set to 60 for all iterations of the model.
Share of DG wait start: To enable that capacity is allocated to each DG according to their respective share of the total number of patients “held” in the Wait_Initiation stock.
Calculated from the equation:
IF (Wait_Initiation)>0 AND SUM (Wait_Initiation)>0 THEN Wait_Initiation/ SUM (Wait_Initiation) ELSE 0

Generalized main operative step (stage PET chosen as an example):

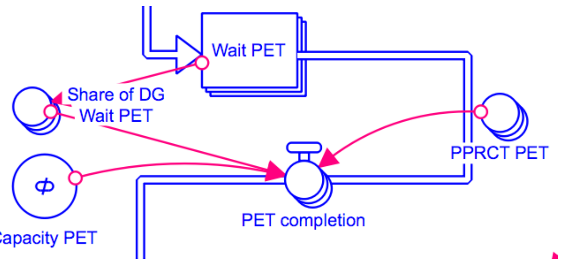

Wait PET: Number of patients waiting for PET. Calculated from the equation: Wait_PET[DG_prep](t) = Wait_PET[DG_prep](t - dt) + (Mould_completion[DG_prep] - PET_completion[DG_prep]) * dt

Where Where DG_prep is a defined structure (array) enabling parallel calculation with unique data for each diagnose group, t=time, dt=delta time (amount of time between calculations in the model).

PPRCT PET: The DG-specific demand for this operative step (PET) expressed as the percentage of patients for whom the operative step has been executed. Some patients may require more than one appointment, hence the value can exceed 100%.
Capacity PET: A selected constant value chosen to not build queues allocated to each DG. Values for all operative steps:
Capacity Mould: 12.8 patients/week
Capacity PET: 1.8 patients/week
Capacity CT: 32.0 patients/week
Capacity MRI: 9.0 patients/week
Capacity Target Definition: 31.0 patients/week
Capacity Treatment Planning: 36.0 patients/week
Capacity QA: 12.5 patients/week

Share of DG Wait PET: Ensures that capacity is allocated to each DG according to their share of the total number of patients "held" in the Wait PET stock. Calculated from the equation:
IF SUM (Wait_PET)>0 AND Wait_PET >0 THEN Wait_PET/ SUM (Wait_PET) ELSE 0
PET completion: Main operative step that enables the flow of patients through the PET stage. It allocates available total capacity for the step proportionally to each DG through Share of DG Wait PET (waiting patients of each DG) and considers the share of patients of each DG that is actually handled in the step. By division with PPRCT_PET (percentage of patients within a DG that is utilizing PET) the flow of patients corresponds to some being handled in the step and some not. If PPRCT_PET has a low value, a larger number of patients in the DG quickly pass through the step and vice versa.
Calculated from the equation: IF PPRCT_PET>0 THEN Share_of_DG_Wait_PET* Capacity_PET/(PPRCT_PET/100) ELSE 0
Accumulation of patients ready to start treatment: The final step were patients ready for treatment accumulates. Calculated from the equation:
Accumulation_of_patients_ready_to_start_treatment[DG_prep](t) = Accumulation_of_patients_ready_to_start_treatment[DG_prep](t - dt) + (QA_completion[DG_prep]) * dt {NON-NEGATIVE}
Where Where DG_prep is a defined structure (array) enabling parallel calculation with unique data for each diagnose group, t=time, dt=delta time (amount of time between calculations in the model).

Model settings

Integration method were 2nd-order Runge-Kutta and delta time were 0.1. All accumulations are initiated with a value of zero.
